# Arginine and Spermine Ameliorate Water Deficit Stress in Fenugreek (*Trigonella foenum-graecum* L.) by Enhancing Growth and Physio-Biochemical Processes

**DOI:** 10.3390/antiox14030329

**Published:** 2025-03-11

**Authors:** Ali A. Badawy, Wadha Kh. Alshammari, Noura F. G. Salem, Woroud S. Alshammari, Hebat-Allah A. Hussein

**Affiliations:** 1 Botany and Microbiology Department, Faculty of Science, Al-Azhar University, Nasr City, Cairo 11884, Egypt; 2 Biology Department, College of Science, University of Hafr Al Batin, Hafr Al-Batin 31991, Saudi Arabia; 3 Botany and Microbiology Department, Faculty of Science (Girls Branch), Al-Azhar University, Cairo 11754, Egypt; 4 Department of Science and Technology, University College at Nairiyah, University of Hafr Al Batin, Nairiyah 31981, Saudi Arabia

**Keywords:** growth regulators, fenugreek, antioxidants, osmolytes, yield traits, drought stress

## Abstract

Plants face various stresses, particularly water deficit, which negatively impacts photosynthesis, growth, and development, thereby limiting agricultural production. Utilizing growth regulators, such as amino acids and polyamines, to enhance osmotic stress tolerance is a crucial area of research in sustainable agriculture. This study investigates the impact of arginine and spermine treatments on various growth attributes, enzymatic and non-enzymatic antioxidants, photosynthetic pigments, protein and lipid peroxidation, and yield traits of fenugreek plants under both normal and drought conditions. The results indicate that drought conditions significantly reduce morphological characteristics, leaf pigments, and yield traits. However, the application of arginine and spermine enhances these parameters, with spermine showing a more pronounced effect. Additionally, treatments boost antioxidant enzymes activities and improve the levels of non-enzymatic antioxidants and osmolytes, contributing to better stress tolerance and growth performance. Principal component analysis confirms that drought significantly alters plant physiology, increasing proline and malondialdehyde levels, while arginine and spermine alleviate drought stress by enhancing antioxidant activity and osmolyte accumulation. The current investigation aims to evaluate the effectiveness of spermine and arginine treatments on various growth attributes and stress tolerance of fenugreek plants under normal and drought conditions, focusing on their comparative efficacy.

## 1. Introduction

Fenugreek (*Trigonella foenum-graecum* L.) is a versatile annual herb from the Fabaceae family, extensively grown in India and across the Mediterranean region, Northern Africa, China, parts of Europe, Australia, and recently North America [1]. Traditionally used as a food and medicine, fenugreek seeds are incorporated into wheat and maize flour in Egypt and serve as a spice, a vegetable, forage for cattle, and for medicinal purposes [2]. The plant is rich in biochemical constituents such as steroids, saponins, polysaccharides, alkaloids, volatile oils, fixed oil, proteins, sugars, mucilage, and flavonoids, which contribute to its medicinal and pharmaceutical significance [3]. Its known for its immunological, anticarcinogenic, antioxidant, antidiabetic, and hypocholesterolemic activities, making it valuable in treating ailments like diabetes and hyperglycemia [4]. Additionally, it is a rich source of protein, lysine, essential nutrients, dietary fiber, and steroid saponins, which are commercially useful for steroid hormone synthesis [5]. Fenugreek’s diverse applications and health benefits underscore its importance as a medicinal and economical plant.

Plant growth and productivity are significantly impacted by various abiotic and biotic stress factors, including low temperature, salt, drought, flooding, heat, oxidative stress, heavy metal toxicity, and pathogens [6,7,8,9,10,11,12]. Drought stress, in particular, poses a major challenge, affecting approximately one-third of the potentially viable land globally due to inadequate water supply. This stress leads to various physiological and biochemical effects on plants, disrupting growth, metabolism, development, productivity, and molecular expressions [13,14,15]. It induces disturbances in the photosynthetic process and carbon metabolism and causes partial stomatal closure, reducing carbon dioxide availability and causing imbalances in nitrogen and carbon metabolism [16]. The upcoming water shortage in certain regions around the world presents a significant challenge to agricultural development and crop production. Under drought conditions, plants store osmolytes like sugars and amino acids to regulate water uptake [17]. Sugars are more efficient than proline in replacing water, forming a hydration shell around biomolecules. Proteins are essential for all physiological activities in plant cells, and their levels rise under drought stress [18]. Drought also increases phenols and flavonoids in plants, enhancing their stress tolerance [19]. Stress-induced reactive oxygen species (ROS) deactivate enzyme functionality and cause oxidative disruption to lipids, proteins, and nucleic acids [20], leading to disruptions in water relationships and membrane stability [21]. Also, it has been shown that fenugreek plants experienced significant reductions in growth, photosynthetic pigments, and proteins under water stress [5].

Plants possess various methods to face environmental stressors and enhance their physiological activity. One effective strategy is the application of chemical compounds, such as plant growth regulators like amino acids and polyamines. These regulators are not only cost-effective but also play a crucial role in boosting plant stress tolerance.

L-arginine is a highly versatile amino acid in living cells, serving as a constituent of proteins and a precursor for the biosynthesis of polyamines, proline, agmatine, and cell signaling molecules like nitric oxide and glutamine [22]. It plays a fundamental role in stress tolerance due to its involvement in numerous physiological processes, including protein synthesis, osmotic potential, stomatal activity, and vegetative growth [23,24]. Investigations have confirmed that L-arginine is a vital modulator in various processes within higher plants and in their response to stress factors like salinity, water deficiency, and disease [25,26,27]. Arginine is particularly important in nitrogen metabolism in germinating seeds and developing seedlings [24]. It is widely used to increase growth, chemical constituents, and yields of crops. Arginine induces enzymes responsible for antioxidation, such as ascorbate peroxidase, superoxide dismutase, and glutathione reductase, thereby alleviating several stressors effects [28].

Polyamines, such as spermine, spermidine, and putrescine, are low-molecular-weight aliphatic amines found in plant cells [29]. They are essential for various fundamental processes, including cell division, differentiation, root elongation, floral development, leaf senescence, fruit ripening, protein translation, transcript expression, and chromatin organization [30]. Several studies have clarified the essential role of polyamines in enhancing plant tolerance to abiotic stresses. They protect against oxidative damage by preventing lipid peroxidation and neutralizing free radicals [31,32]. Spermine, in particular, modulates several physio-biochemical processes to reduce oxidative damage and enhance plant protection against multiple abiotic and biotic stresses [33]. It acts as a secondary messenger in signaling pathways, regulating plant development and boosting tolerance mechanisms [34]. Spermine applications on various plants have been found to positively impact drought responses by boosting osmolyte accumulation, elevating free polyamine levels, and regulating polyamine biosynthetic genes [35].

The aim of this study is to evaluate the effects of arginine and spermine treatments on the growth attributes, enzymatic and non-enzymatic antioxidants, pigments, osmolytes, and yield traits of fenugreek plants under both normal and drought conditions. This study seeks to determine how these treatments can mitigate the adverse effects of drought stress and enhance plant performance, with a particular focus on the comparative efficacy of arginine and spermine.

## 2. Materials and Methods

### 2.1. Layout of the Experiment

The experiment was carried out at the Botanical Research Station, Botany and Microbiology Department, Faculty of Science, Al-Azhar University, Cairo, Egypt. Seeds of the fenugreek, Giza 3 variety, obtained from the Institute of Crops Research, Agricultural Research Centre, Giza, Egypt, were utilized. Thirty fenugreek seeds were sown in 40 cm diameter earthenware pots containing 7 kg of soil. These pots were divided into two groups: control (unstressed) and drought-stressed (induced by PEG-stimulated drought stress). Each group was further divided into three sub-groups: control (untreated), foliar-treated with 1 mM arginine, and foliar-treated with 1 mM spermine. Seven plants remained in each pot after thinning and were irrigated as needed. Arginine and spermine were applied as a foliar spray twice, on the 25th and 35th days after sowing. Randomized plant samples were harvested on the 45th day after sowing for morphological, biochemical, and physiological analysis.

### 2.2. Plant Lengths and Biomass

Five fenugreek plants from each group were randomly collected to measure growth characteristics, including shoot length (cm), root length (cm), number of leaves, fresh and dry weights of shoots (g), and fresh and dry weights of roots (g).

### 2.3. Enzymatic Antioxidants

Fenugreek buds were used to extract superoxide dismutase (SOD), peroxidase (POD), polyphenol oxidase (PPO), and catalase (CAT), as described by [36]. SOD activity was determined using the method of Marklund and Marklund [37], which involves measuring pyrogallol reduction at 325 nm. POD activity was measured using the method outlined by Bergmeyer [38], focusing on the increase in pyrogallol absorbance at 470 nm. PPO activity was assessed using the method of Matta [39], which measures changes in catechol absorbance at 495 nm. CAT activity was estimated by measuring the cleavage of hydrogen peroxide following the technique of Aebi [40], with spectrophotometric readings at 240 nm.

### 2.4. Non-Enzymatic Antioxidants and Osmolytes

Free proline was determined using the method of Bates et al. [41], with proline levels measured at 520 nm. Total soluble sugars were determined following the described method in [42], with absorbance measured at 620 nm. Phenolic compounds were determined using the procedure in [43], with absorbance measured at 725 nm. Free amino acids were extracted and estimated using a series of standard solutions and a ninhydrin reagent, with absorbance measured at 570 nm [44].

### 2.5. Leaf Pigments

Chlorophylls and carotenoids in fenugreek leaves were quantified according to the described method by Vernon and Seely [45], with absorbance readings taken at 470 nm, 649 nm, and 665 nm.

### 2.6. Proteins and Malondialdehyde

Total soluble proteins were assayed using the technique in [46], with absorbance measured at 750 nm. Malondialdehyde (MDA) was estimated using the method in [47], with absorbance read at 532 nm, 600 nm, and 450 nm.

### 2.7. Yield

At the yield stage, five fenugreek plants were randomly selected to assess yield attributes, including weight of pods/plant, number of pods/plant, weight of seeds/plant, number of seeds/plant, and 100-seed weight.

### 2.8. Statistical Analysis

A two-way ANOVA followed by Tukey’s test was conducted to assess the significance among treatments at a significance level of α = 0.05, ensuring data normality and homogeneity of variances using Shapiro–Wilk’s and Levene’s Median tests, respectively [48]. When assumptions were violated, data transformations were applied. Principal component analysis (PCA) was carried out to illustrate the relationship between treatments and physiological parameters using PC-ORD version 5.

## 3. Results

### 3.1. Morphological Attributes

The provided results in Table 1 illustrate the response of various morphological traits of fenugreek plants, including root length, root fresh weight, root dry weight, shoot length, shoot fresh weight, shoot dry weight, and number of leaves, to arginine and spermine treatments under both normal and drought conditions.

Subjecting fenugreek plants to drought conditions caused notable reductions in various morphological characteristics. Specifically, there were decreases of approximately 12.5% in root length, 33.05% in root fresh weight, 50% in root dry weight, 13.71% in shoot length, 31.32% in shoot fresh weight, 36.15% in shoot dry weight, and 29.33% in the number of leaves, compared to plants under normal conditions.

The treatment with arginine and spermine resulted in noticeable improvements in the measured morphological parameters of fenugreek plants under normal conditions. These improvements included increases of approximately 0.8% and 2.7% in root length, 8.15% and 9.01% in root fresh weight, 25% and 41.67% in root dry weight, 13.31% and 17.74% in shoot length, 16.39% and 60.27% in shoot fresh weight, 12.21% and 41.78% in shoot dry weight, and 14.67% and 34.66% in the number of leaves in response to arginine and spermine, respectively.

Both under normal and adverse (drought) conditions, treating fenugreek plants with arginine and spermine showed significant responses in the morphological growth parameters. These included improvements of approximately 5% and 9.8% in root length, 14.1% and 28.21% in root fresh weight, 50% and 94.44% in root dry weight, 9.35% and 11.68% in shoot length, 35.64% and 43.32% in shoot fresh weight, 38.97% and 54.78% in shoot dry weight, and 32.08% and 33.96% in the number of leaves, respectively, when compared with untreated plants under stress.

### 3.2. Enzymatic Antioxidants

**Figure 1:**
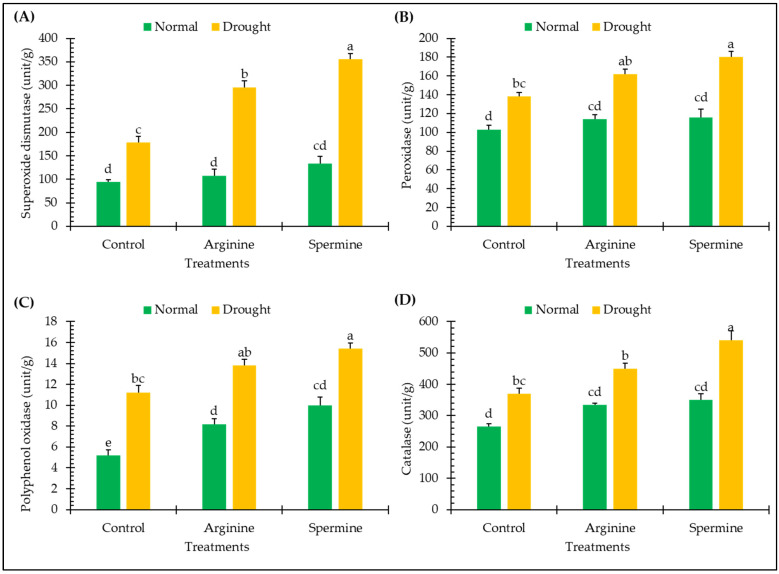
Effect of arginine and spermine treatments on the activities of superoxide dismutase (**A**), peroxidase (**B**), polyphenol oxidase (**C**), and catalase (**D**) in fenugreek plants under normal and drought conditions. Bars are expressed as the mean ± the standard error. Distinct letters denote significant variations among the means.

The data presented in Figure 1 demonstrate how different antioxidant enzymes of fenugreek plants, such as superoxide dismutase, peroxidase, polyphenol oxidase, and catalase, respond to treatments with arginine and spermine under both normal and drought conditions.

The activities of enzymes of fenugreek plants, such as superoxide dismutase, peroxidase, polyphenol oxidase, and catalase, boosted significantly by approximately 89.36%, 33.98%, 115.38%, 39.62%, respectively, when subjected to drought conditions, as opposed to normal conditions.

Administering arginine and spermine to fenugreek plants led to substantial inductions in the activities of various enzymatic antioxidants under normal conditions. Specifically, superoxide dismutase increased by approximately 14.89% and 42.55%, peroxidase increased by 10.68% and 12.62%, polyphenol oxidase increased by 57.69% and 92.31%, and catalase increased by 26.42% and 32.08%, respectively, in response to arginine and spermine.

Moreover, under drought conditions, fenugreek plants treated with arginine and spermine exhibited notable improvements in the activities of various antioxidant enzymes. These included enhancements of approximately 66.29% and 100% in superoxide dismutase, 17.39% and 30.43% in peroxidase, 23.21% and 37.5% in polyphenol oxidase, and 21.62% and 45.95% in catalase, respectively, in comparison with untreated plants under stress.

### 3.3. Non-Enzymatic Antioxidants and Osmolytes

**Figure 2:**
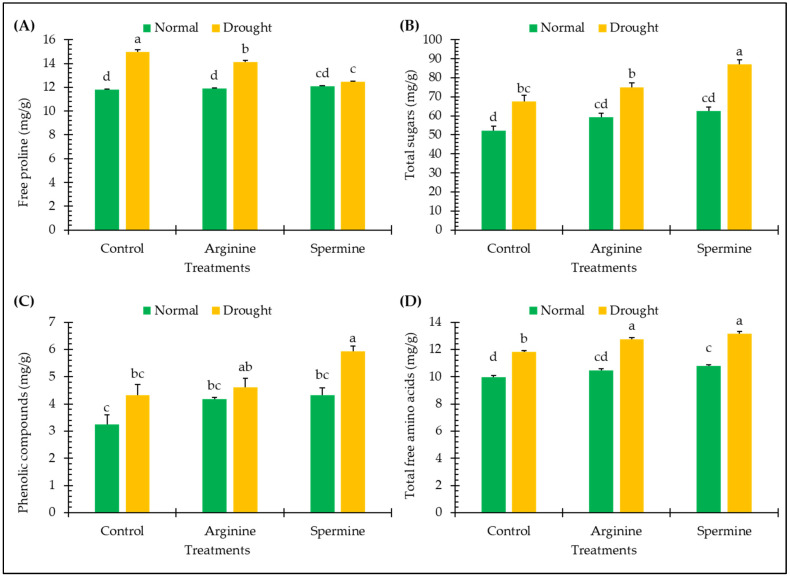
Effect of arginine and spermine treatments on the levels of free proline (**A**), total sugars (**B**), phenolic compounds (**C**), and total free amino acids (**D**) in fenugreek plants under normal and drought conditions. Bars are expressed as the mean ± the standard error. Distinct letters denote significant variations among the means.

The findings in Figure 2 illustrate the impact of arginine and spermine treatments on various non-enzymatic antioxidants and osmolytes of fenugreek plants, such as sugars, proline, amino acids, and phenolics, under both normal and drought conditions.

Under drought conditions, fenugreek plants exhibited significant promotions in the levels of free proline, total sugars, phenolic compounds, and total free amino acids by approximately 26.84%, 29.16%, 33.57%, and 18.38%, respectively, in comparison to plants grown under normal conditions.

Application of arginine and spermine resulted in noticeable improvements in the levels of free proline, total sugars, phenolic compounds, and total free amino acids in fenugreek plants under normal conditions. These enhancements included increases of approximately 0.92% and 2.43% in proline, 13.33% and 19.63% in sugars, 29.18% and 33.38% in phenolics, and 5% and 8.23% in amino acids, respectively, in response to arginine and spermine.

Treating fenugreek plants with arginine and spermine showed significant variations in the abovementioned attributes not only under normal conditions but also under unfavorable (drought) conditions. These included decreases of approximately 5.55% and 16.79% in proline levels and increases of approximately 11.06% and 28.81% in sugars, 6.77% and 37.06% in phenolics, and 7.96% and 11.21% in amino acids, respectively, compared to untreated plants under stress.

### 3.4. Photosynthetic Pigments

**Figure 3:**
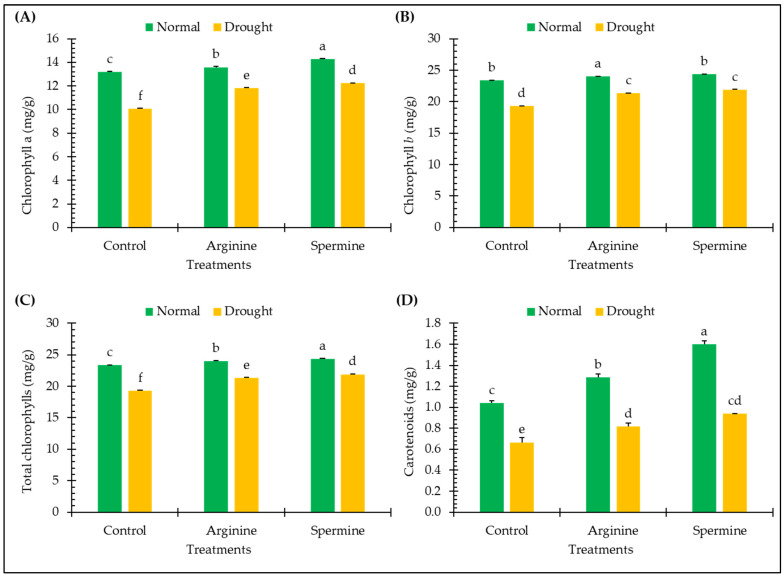
Effect of arginine and spermine treatments on the contents of chlorophyll *a* (**A**), chlorophyll *b* (**B**), total chlorophylls (**C**), and carotenoids (**D**) in fenugreek plants under normal and drought conditions. Bars are expressed as the mean ± the standard error. Distinct letters denote significant variations among the means.

Figure 3 presents the effects of arginine and spermine treatments on certain leaf pigments of fenugreek plants, including chlorophyll *a*, chlorophyll *b*, total chlorophylls, and carotenoids, in normal and drought conditions.

Drought exposure resulted in substantial decreases in the levels of photosynthetic pigments of fenugreek leaves, including chlorophyll *a* (23.60%), chlorophyll *b* (9.69%), total chlorophylls (17.52%), and carotenoids (36.06%), in comparison to plants under normal conditions.

The treatment of fenugreek plants with arginine and spermine under normal conditions led to slight enhancements in leaf pigments especially in carotenoids which were promoted by approximately 23.69% and 53.7% in carotenoids. Furthermore, treating the tested plants with arginine and spermine resulted in significant increases of approximately 17.37% and 21.45% in chlorophyll *a*, 3.18% and 4.92% in chlorophyll *b*, 10.59% and 13.55% in total chlorophylls, and 22.67% and 41.14% in carotenoids, respectively, compared to untreated plants under stress.

### 3.5. Proteins and Malondialdehyde

**Figure 4:**
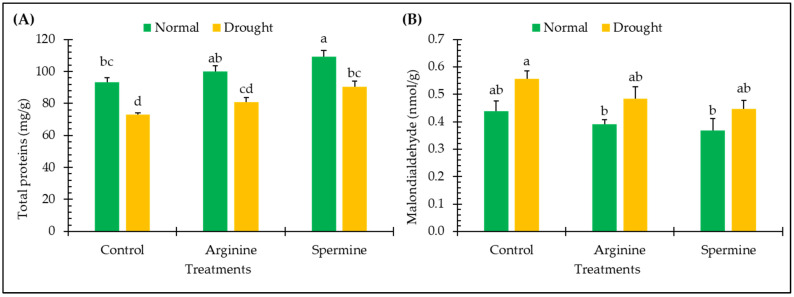
Effect of arginine and spermine treatments on the amounts of total proteins (**A**) and malondialdehyde (**B**) in fenugreek plants under normal and drought conditions. Bars are expressed as the mean ± the standard error. Distinct letters denote significant variations among the means.

The results displayed in Figure 4 highlight the impact of arginine and spermine treatments on the amounts of total proteins and malondialdehyde in fenugreek plants in both normal and drought conditions.

The drought stress led to notable variations, with reductions of about 21.68% in total proteins contents, while increments of about 27.06% were observed for the malondialdehyde contents of fenugreek plants, relative to unstressed plants.

Remarkable enhancements of 6.98% and 17.19% for protein contents and noticeable inhibitions of 10.87% and 16.02% for malondialdehyde levels in fenugreek plants were observed under normal conditions following treatment with arginine and spermine. Furthermore, under drought conditions, fenugreek plants treated with arginine and spermine showed obvious accumulations in protein amounts by about 10.55% and 23.91%, while observed suppressions in malondialdehyde contents (as an indicator for the suppression of lipid peroxidation) reached about 12.84% and 19.55%, respectively, compared to untreated plants under stress.

### 3.6. Yield Attributes

The data in Table 2 show how fenugreek plants’ yield indices, such as pod weight, pod number, seed weight, seed number, and 100-seed weight, are affected by arginine and spermine treatments under both normal and drought conditions.

When exposed to drought conditions, fenugreek plants showed significant declines in yield traits, such as weight of pods/plant, number of pods/plant, weight of seeds/plant, number of seeds/plant, and 100-seed weight, by approximately 34.72%, 27.08%, 30.51%, 24.55%, and 18.18%, respectively, compared to those under normal conditions.

The application of arginine and spermine led to noticeable improvements in the yield characteristics of fenugreek plants under normal conditions. These enhancements included increases of approximately 29.27% and 36.63% in the weight of pods, 22.91% and 75% in the number of pods, 41.2% and 64.75% in the weight of seeds, 30.9% and 41.14% in the number of seeds, and 11.9% and 24.69% in the 100-seed weight, respectively, in response to arginine and spermine.

Both under normal and adverse (drought) conditions, treating fenugreek plants with arginine and spermine showed significant responses in yield parameters. These included increases of approximately 12.48% and 29.64% in the weight of pods, 11.43% and 27.14% in the number of pods, 14.95% and 28.16% in the weight of seeds, 14.57% and 23.99% in the number of seeds, 13.17% and 21.81% in the 100-seed weight, respectively, compared to untreated plants under stress.

### 3.7. Principal Component Analysis

**Figure 5:**
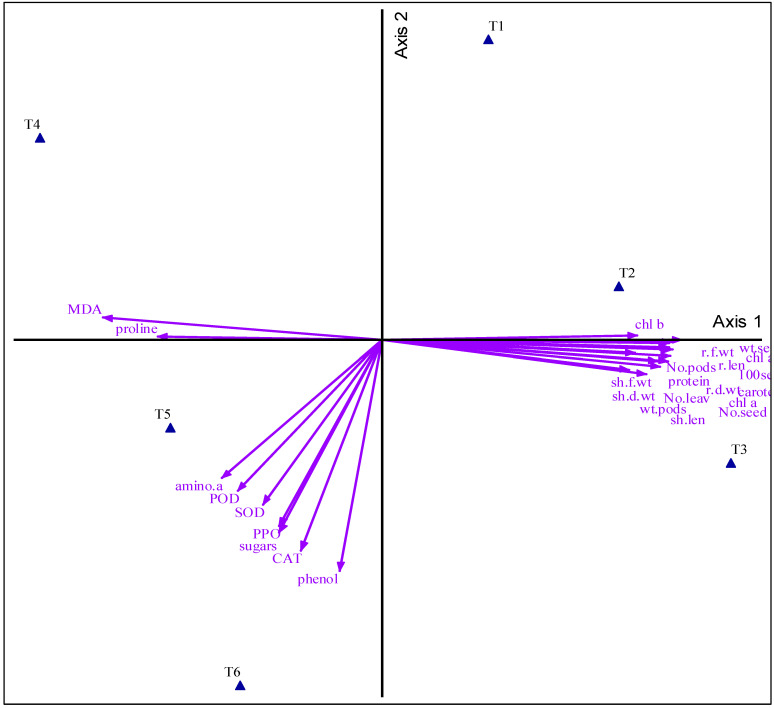
Principal component analysis visualizes the relationships of fenugreek growth attributes with treatments. Different abbreviations used in the chart are as follows: no treatment under normal irrigation conditions (control) (T1), arginine without drought (T2), spermine without drought (T3), drought (T4), arginine with drought (T5), spermine with drought (T6), r.len (root length), r.f.wt (root fresh weight), r.d.wt (root dry weight), sh.len (shoot length), sh.f.wt (shoot fresh weight), sh.d.wt (shoot dry weight), No.leav (number of leaves), wt.pods (weight of pods), No.pods (number of pods), wt.seed (weight of seeds), No.seed (number of seeds), 100 seed (100-seeds weight), chl a (chlorophyll *a*), chl b (chlorophyll *b*), chl a + b (total chlorophyll), caroten (carotenoids), protein, amino.a (amino acids), proline, phenol (phenolic compounds), sugars, MDA (malondialdehyde), SOD (speroxide dismutase), POD (peroxidase), PPO (polyphenol oxidase), and CAT (catalase).

The provided image (Figure 5) from the PCA plot shows distinct clustering of treatments, with drought-stressed treatments (T4, T5, and T6) clearly separated from non-drought treatments (T1, T2, and T3), indicating different physiological responses. Drought alone (T4) induces unique physiological responses, while arginine (T5) and spermine (T6) treatments mitigate drought effects, leading to similar but distinct profiles compared to untreated plants. Non-drought treatments cluster closely, suggesting minimal changes in plant physiology under normal conditions. Physiological parameters such as proline and malondialdehyde (MDA) are strongly associated with drought-induced stress (T4), while antioxidant enzymes (POD, SOD, and CAT), sugars, phenols, and amino acids are linked to T5 and T6, highlighting their role in mitigating drought stress. Growth-related parameters are associated with non-drought treatments, reflecting better growth performance under normal irrigation.

## 4. Discussion

Enhancing plant growth, development, productivity, and resistance to climatic stress are crucial areas in agriculture and plant-based biotechnologies [49]. Throughout their life cycle, plants face numerous biotic and abiotic stresses, with drought being one of the most severe, leading to significant reductions in agricultural productivity and posing a threat to global food security [50,51]. Drought stress affects plants on both the morphological and molecular levels, decreasing growth and productivity [13]. Addressing plant drought tolerance is a major challenge in modern agriculture, where biostimulants, like amino acids and polyamines, play a vital role [52]. These substances help mitigate the harmful effects of stress and offer essential protection against oxidative damage, ultimately enhancing plant development and productivity [53].

In the current study, drought stress significantly reduced the number of fenugreek morphological parameters, including plant height, root depth, fresh and dry weights of shoots and roots, and the number of leaves. This aligns with previous studies on various crops, showing decreased germination and growth under drought stress [54,55,56,57]. The growth inhibition is attributed to reduced cell turgor, suppressing cell elongation and development, and tissue water loss, hindering cell division and elongation [58,59]. Arginine is a crucial amino acid that significantly contributes to plant growth. Research indicates that its application results in notable enhancements in the morphological growth attributes of various crops [28,60,61]. Our results indicate that foliar application of arginine mitigates drought effects on fenugreek plant growth. Arginine may enhance plant responses to drought stress through its conversion into proline and nitric oxide, which are essential for drought adaptation. Furthermore, arginine’s ability to counteract abiotic stresses could be linked to the production of polyamines, which play significant roles in various biological processes such as growth, metabolism, and stress responses [26,27]. Similarly, the application of spermine in this study resulted in marked improvements in the growth indices of fenugreek under normal or drought conditions, consistent with previous findings on other crops [62,63,64]. Spermine plays a significant role in cell division, elongation, and protein synthesis [65]. It is particularly involved in shoot and root development, floral induction, fruit set, leaf senescence, DNA synthesis, osmolyte balance, chlorophyll protection, gene transcription, and protein translation [62,66]. Spermine plays a vital role in enabling plants to effectively respond to environmental stresses, including drought [35,67].

The activation of enzymatic antioxidants is essential for mitigating stress [68,69]. Enzymatic antioxidant activities increase when mung bean plants face water deficit conditions [70]. Additionally, previous investigations reported that drought stress induces antioxidant enzyme activities in various plants [71,72,73], aligning with our findings. Enhanced antioxidant enzyme activities under water stress are attributed to elevated hydrogen peroxide and singlet oxygen levels. In response, plants typically elevate their antioxidant activities to scavenge reactive oxygen species and mitigate stress [74]. Arginine pretreatment increased enzyme activity in different plants under normal and stress conditions [61,75]. Arginine, an amino acid, plays a significant role in alleviating drought stress [76]. Its application promotes enzyme activity, aiding in converting free radicals into water and oxygen, protecting the cell [26]. Similarly, spermine treatment ameliorated drought-induced osmotic stress by increasing catalase, superoxide dismutase, peroxidase, and polyphenol oxidase activities in several crops [14,77]. Enhanced antioxidant activities resulting from polyamine treatments are associated with improved molecular signaling, which supports adaptive plant responses to water stress [70]. Earlier studies indicate that polyamines help stabilize membranes and neutralize free radicals by boosting antioxidant activities [31].

The current study demonstrated that water scarcity in fenugreek plants led to the accumulation of certain osmolytes and non-enzymatic antioxidants, including proline, sugars, phenolics, and amino acids. These findings align with several studies reporting high levels of osmo-protectants in different crops under drought stress [78,79,80,81]. Additionally, the results showed that arginine and spermine applications increased the content of amino acids, sugars, and phenolics while reducing proline levels, as previously documented in various investigations [32,61,76,82]. The accumulation of these biomolecules serves as a tolerance strategy to reduce the oxidative damage caused by stress. Increases in soluble sugars, proline, and free amino acids in stressed plants help the cells adapt to drought conditions [83,84]. These osmolytes can scavenge free radicals, inhibit cellular redox potential, adjust osmotic pressure, stabilize membranes and proteins, and maintain the relative water content necessary for plant growth and metabolism [6,85,86]. It is noteworthy that proline levels decreased when drought-stressed plants were treated with arginine and spermine, indicating reduced plant sensitivity to drought. This indicates that polyamines and their precursor arginine play crucial roles as modulators in higher plants, influencing growth, physiological processes, development, and responses to stress factors [24,87].

Consistent with our findings, drought stress led to a reduction in chlorophyll and carotenoid levels in cotton [88], wheat [89], peanut [79], barley [28], and rice [90] and other important crops. Drought conditions have been reported to damage the photosynthetic system, reduce gas exchange, and decrease growth parameters and productivity [91]. The decline in the net photosynthetic rate under drought stress is due to biochemical disruptions caused by lipid oxidation and protein denaturation, which are crucial for pigment and chloroplast structures [92]. Conversely, the application of arginine to both normal and stressed fenugreek plants was found to significantly enhance leaf pigment content. Our findings align with previous studies on different plants [60,61,78]. The role of arginine in boosting pigment content can be attributed to its function as an amino acid that serves as a nitrogen source for chlorophyll formation [23]. The ability of arginine to alleviate stress and enhance growth characteristics is likely due to the production of polyamines, which participate in various biological processes such as growth, development, and responses to abiotic stresses [26]. Moreover, spermine greatly enhances the biosynthesis of chlorophyll pigments and PSII function through stomatal regulation, modulation of electron transfer chains to PSI receptors, and improvement in CO_2_ assimilation rates, plant growth, and biomass yield under stress conditions [93]. This enhancement is linked to the increased stability of thylakoid membranes, plastid biogenesis, and the prevention of chlorophyll degradation [94,95]. Additionally, spermine promotes chlorophyll synthesis by increasing the uptake of magnesium ions, which are essential components of chlorophyll [96].

In the current investigation, drought conditions led to a reduction in protein content and an increase in malondialdehyde (a product of membrane lipid peroxidation). These findings are consistent with studies on maize [97], barley [55], wheat [62], lettuce [67], and soybean [98], regarding lipids, as well as fenugreek [5], soybean [33,98], and cowpea [99], regarding proteins, under various stress conditions. Under stress, plants may enhance proteolytic enzymes, leading to protein degradation, and accumulate excessive reactive oxygen species (ROS), which destabilize cell membranes and cause damage to DNA, pigments, proteins, and lipids [64,100]. The inhibitory effects of drought may be linked to reduced photosynthesis, as carbohydrates, the primary photosynthetic product, are essential for forming vital biomolecules [101]. Supplementing fenugreek plants, whether under natural or stress conditions, with arginine or spermine enhances protein content and reduces malondialdehyde accumulation from lipid oxidation, as documented in previous studies [27,75,77,97]. L-arginine plays a crucial role in physiological processes by modulating polypeptides involved in oxidative stress. It contributes to polyamine synthesis, membrane stability, osmotic balance, signal transduction, and electron transport [25,102]. Arginine’s role in counteracting abiotic stresses may involve polyamine production, which supports growth, metabolism, and stress responses [26]. Spermine also acts a substantial role in post-transcriptional protein modifications, stabilizing protein conformation and function [103]. Exogenous spermine application alleviates stress effects by reducing lipid peroxidation and increasing total polyphenols, catalase, and superoxide dismutase activities [64,104]. Polyamines are essential for protein homeostasis, ROS detoxification, and antioxidative machinery activation under stress conditions [66]. They maintain membrane stability and permeability, enhance catalase activity, and reduce H_2_O_2_ content, ROS markers, and lipid peroxidation, thereby providing broad-spectrum tolerance against various stresses [62,105].

A water deficit significantly reduces yield attributes in crops such as tomatoes [106], wheat [107,108], and cotton [80] by negatively impacting growth and productivity. The reduction in growth and yield is associated with the excessive production of reactive oxygen species, which cause damage to cell membranes and components [97,109]. However, the application of arginine significantly increases plant yield under drought stress by promoting protein, proline, and polyamine biosynthesis, enhancing stomatal activity, osmotic potential, and overall growth [23,110]. Amino acids also provide essential substances for protein formation and function as osmo-regulators, increasing cellular osmotic components [31,111]. In this respect, polyamines play a crucial role in physiological processes such as reproductive organ development, tuberization, floral initiation, fruit development, and ripening [66], in addition to their role in maintaining turgor pressure [70,80].

PCA analysis confirms that drought significantly alters plant physiology, increasing proline and MDA levels. Arginine and spermine effectively alleviate drought stress by enhancing antioxidant activity and osmolyte accumulation, supporting their role as protective agents. These substances have a vital impact, highlighting their potential as targeted treatments for enhancing crop resilience to water deficit stress.

## 5. Conclusions

Applying arginine and spermine has been shown to effectively alleviate the adverse effects of drought stress on fenugreek plants. These treatments enhance both morphological growth characteristics and yield traits, while also improving the activities of antioxidant enzymes, as well as the levels of non-enzymatic antioxidants and osmolytes. Notably, spermine exhibits greater efficacy in promoting growth and stress tolerance. This study underscores the potential of utilizing arginine and spermine as eco-friendly and cost-effective solutions for enhancing plant performance under both normal and drought conditions.

## Figures and Tables

**Table 1:** Effect of arginine and spermine treatments on the morphological growth characteristics of fenugreek plants under normal and drought conditions. Values are expressed as mean ± standard error. Distinct letters denote significant variations among the means.

	Treatments	Control	Arginine	Spermine
Conditions				
**Root length (cm)**				
Normal		12.8 ± 0.44 ab	12.9 ± 0.43 ab	13.14 ± 0.48 a
Drought		11.2 ± 0.60 b	11.76 ± 0.57 ab	12.3 ± 0.48 ab
**Root fresh weight (g)**				
Normal		0.47 ± 0.014 a	0.504 ± 0.016 a	0.508 ± 0.021 a
Drought		0.31 ± 0.021 c	0.356 ± 0.013 bc	0.4 ± 0.02 b
**Root dry weight (g)**				
Normal		0.07 ± 0.008 bc	0.09 ± 0.007 ab	0.10 ± 0.006 a
Drought		0.04 ± 0.007 d	0.05 ± 0.007 cd	0.07 ± 0.006 bc
**Shoot length (cm)**				
Normal		24.8 ± 0.48 a	28.1 ± 1.01 a	29.2 ± 1.03 a
Drought		21.4 ± 1.05 a	23.4 ± 1.13 a	23.9 ± 0.88 a
**Shoot fresh weight (g)**				
Normal		5.64 ± 0.42 bc	6.56 ± 0.39 b	9.04 ± 0.45 a
Drought		3.87 ± 0.23 d	5.25 ± 0.29 c	5.55 ± 0.36 bc
**Shoot dry weight (g)**				
Normal		0.85 ± 0.065 b	0.96 ± 0.062 b	1.21 ± 0.055 a
Drought		0.54 ± 0.067 c	0.76 ± 0.058 b	0.84 ± 0.049 b
**Number of leaves**				
Normal		30 ± 1.68 c	34.4 ± 1.20 b	40.4 ± 1.33 a
Drought		21.2 ± 0.95 d	28 ± 1.08 c	28.4 ± 1.20 c

**Table 2:** Effect of arginine and spermine treatments on the yield characteristics of fenugreek plants under normal and drought conditions. Values are expressed as the mean ± the standard error. Distinct letters denote significant variations among the means.

	Treatments	Control	Arginine	Spermine
Conditions				
**Weight of pods/plant (g)**				
Normal		6.09 ± 0.27 ab	7.87 ± 0.31 a	8.32 ± 0.28 a
Drought		3.97 ± 0.20 c	4.47 ± 0.41 c	5.15 ± 0.38 ab
**Number of pods/plant**				
Normal		19.2 ± 1.11 c	23.6 ± 1.20 b	33.6 ± 0.66 a
Drought		14 ± 1.08 c	15.6 ± 0.88 de	17.8 ± 1.25 cd
**Weight of seeds/plant (g)**				
Normal		3.97 ± 0.22 b	5.6 ± 0.27 a	5.82 ± 0.30 a
Drought		2.76 ± 0.33 c	3.17 ± 0.29 bc	3.53 ± 0.23 bc
**Number of seeds/plant**				
Normal		211 ± 8.76 b	276.2 ± 11.54 a	297.8 ± 13.11 a
Drought		159.2 ± 8.83 c	182.4 ± 13.30 bc	197.4 ± 5.94 b
**100-seeds weight (g)**				
Normal		1.78 ± 0.08 bc	1.99 ± 0.06 ab	2.22 ± 0.07 a
Drought		1.46 ± 0.05 d	1.65 ± 0.11 cd	1.78 ± 0.09 bc

## Data Availability

The data presented in this study are available upon request from the corresponding author.
